# Clinician perspectives on the feasibility of a pain management protocol in kidney transplant

**DOI:** 10.1186/s12882-026-05046-1

**Published:** 2026-05-22

**Authors:** Cassandra B. Iroz, Julie K. Johnson, Vinayak S. Rohan

**Affiliations:** 1 Northwestern University, Feinberg School of Medicine, Chicago, IL USA; 2 University of North Carolina at Chapel Hill, Chapel Hill, NC USA

**Keywords:** Opioids, Pain management, Qualitative research, Pre-implementation, Feasibility

## Abstract

**Background:**

Opioids are often prescribed to treat pain after kidney transplant (KT) but pose significant risks to patients as well as the risk of diversion of unused opioids into the community. While there is growing interest, only 18% of KT programs in the United States have a pain management protocol. We described the current pain management practices in KT at a large academic medical center and assessed the feasibility of implementing a pain management protocol.

**Methods:**

We conducted semi-structured individual interviews and focus groups with clinicians from a large-volume KT program. The interviews and focus groups were audio-recorded, transcribed verbatim, and coded inductively. We used the constant comparative approach to identify and refine emerging themes.

**Results:**

Twenty-three clinicians participated including 7 advanced practice providers, 7 nurses, 5 transplant fellows, 3 attending surgeons, and 1 pharmacist. We identified six overarching themes: (1) clinicians report prescribing opioids to most KT recipients (KTRs) as outlined in the standardized postoperative order set; (2) there is provider variation in opioid and non-opioid prescribing beyond the initial order set; (3) pain management requires a balance of setting realistic expectations about postoperative pain and controlling pain sufficiently for KTRs to complete activities of daily living; (4) clinicians believe that it is feasible to implement a protocol as long as it is flexible to individualize pain management based on patient-reported level and type of pain; (5) an acceptable protocol for KTRs includes preoperative blocks, scheduled acetaminophen, additional non-opioid strategies (e.g., ice, lidocaine patches, muscle relaxants, and gabapentin) and reserves oxycodone for breakthrough pain; (6) barriers to implementing a pain management protocol include concerns about inadequate pain control, delays in treatment, limited resources for preoperative blocks, and challenges changing clinician behaviors.

**Conclusions:**

Although there is variation in current pain management practices, clinicians were enthusiastic about the implementation of a standardized pain management protocol. Clinicians agreed that opioids should be used sparingly but are needed for some KTRs and emphasized the need for individualized pain management. Effective implementation will require coordination with anesthesiologists for preoperative blocks and coordination with nursing and prescribers to ensure timely follow up for breakthrough pain.

**Supplementary Information:**

The online version contains supplementary material available at 10.1186/s12882-026-05046-1.

## Introduction

Opioids are often prescribed to treat pain after kidney transplant but pose significant risks to patients as well as public health risks to the community. Prescription opioids were responsible for over 13,000 overdose deaths in the United States (US) in 2023 [1]. In 2017, costs for opioid use disorder and fatal overdose from prescription and illicit opioids were estimated to be $1.02 trillion USD [2]. Given these data, the opioid epidemic has been categorized as a public health emergency. It is imperative that all healthcare professionals do their part to combat the opioid epidemic by decreasing the number of opioids dispensed and utilizing alternative pain control when clinically appropriate. Opioids have been the primary modality of managing postoperative pain in the US. Patients are often prescribed more opioids than needed to manage their postoperative pain, with research showing that only 28% of the prescribed opioids are utilized by general surgery patients [3]. Unused medication is a reservoir for misuse among patients and relatives. The most recent National Survey on Drug Use and Health found that 42.3% of people who misused prescription pain relievers in the last year obtained them from a friend or relative, compared to only 7.6% who purchased them from a drug dealer or stranger [4]. Surgery can also be a risk factor for opioid misuse as new persistent use of opioids is seen in 5.9% of patients after minor surgery and 6.5% after major surgery [5]. 

The field of solid organ transplantation is in a unique position in the opioid epidemic. Although the number of organs available for transplant has increased due to opioid-related mortality, research has shown that outcomes after transplant are adversely affected by preoperative and postoperative opioid consumption by kidney transplant recipients (KTRs). Our previous study showed that pre-transplantation opioid exposure led to higher rates of readmissions [6]. Abbot et al., found that compared to no or short-term opioid use, long-term use of opioids after kidney transplant was associated with a significant increase in graft loss and death (HR1.33 and 1.61, respectively) [7]. Lentine et al., found that high-level opioid use in the first year after transplant predicted a two-fold increased risk of death and a 68% increased risk of all-cause graft failure, which persisted over five years of observation [8]. 

There is currently no standard approach for managing pain following kidney transplant both in the hospital and after KTRs are discharged home. Kidney transplant is among the most common and standardized transplant operations, creating an ideal environment for developing standardized protocols for pain management. Over the last few years, there has been a national effort in the transplant community to develop pain management protocols to limit opioid use. Pain management protocols in individual transplant centers have shown good short- and long-term outcomes for reduced opioid utilization [9–15]. These protocols have included a combination of techniques, such as education, multimodality pain protocols, and restrictive prescription practices. Despite the success of these protocols in reducing opioid utilization, wider dissemination and adoption have been lacking. A recent survey of kidney transplant programs in the US showed that 82.7% of the surveyed programs did not have any pain management protocols in place [16]. There remains a question of why pain management protocols are not more widely used, barriers to their implementation, and experiences of clinical teams. Qualitative research methods are critically important in implementation science to understand not only if an intervention is effective, but to answer questions of how and why efforts to implement best practices sometimes are successful and other times fail [17, 18]. While previous pain management protocols have been effective, it is unclear why adoption of such interventions is so low. We therefore sought to conduct a qualitative study to access pre-implementation clinician perspectives. In this study, we aimed to describe the current pain management practices and assess the feasibility of implementing a pain management protocol at a large academic medical center.

## Methods

### Setting and participants

We conducted semi-structured interviews and focus groups with attending transplant surgeons, surgeons in the transplant fellowship program (“fellows”), inpatient and outpatient Advanced Practice Providers (APPs) (nurse practitioners and physician assistants), pharmacists, and nurses. We recruited a purposive sample by emailing surgeons, pharmacists, and APPs in the kidney transplant program to participate in the study. For the nursing focus group, the charge nurse for the inpatient transplant unit recruited a group of nurses to participate. All participants were employed at a large volume (approximately 350 kidney transplants per year), urban, academic transplant center. Other pain management and opioid minimization interventions had recently been implemented in general surgery and for enhanced recovery after surgery (ERAS) initiatives, but not within transplant surgery.

### Data collection

The interview topics included how the interviewees discuss, treat, and manage pain for KTRs and the feasibility of implementing a pain management protocol. Participants were shown a draft pain management protocol, based on a quality improvement study at a different institution [11], and asked to provide their opinions and adaptations they believed would be important for local implementation. The interview/focus group guide, including the draft pain management protocol, is provided in the supplementary materials. The guide did not formally change over time, but the use of follow-up questions and prompts varied based on participant responses, as is common for semi-structured interviews [19]. 

The interviews were conducted either in-person or virtually over Zoom (Version 6.3.10), depending on the interviewee’s preference. All participants provided verbal informed consent prior to beginning the interview or focus group. The focus group with the nurses was conducted in the break room and the focus group with the transplant fellows was conducted in a conference space in the hospital with no other individuals present. Interviews and focus groups were on average 23.5 minutes (SD 9.4; range 10.6–45.9). The participants were told that the goal of the project was to understand their current practices and solicit their ideas for improvement. Data collection was completed between May and December 2024.

### Data analysis

Interviews and focus groups were audio-recorded and transcribed verbatim. Transcripts were not returned to participants for comment or verification. The interviewer took brief field notes during and immediately after each interview/focus group. A codebook was developed inductively, and all transcripts were coded by one researcher. The same researcher then conducted thematic analysis to group codes and identify overarching themes, with iterative discussions with the research team. No major disagreements arose and minor differences in interpretation were discussed and resolved through consensus. Formal analysis was conducted after the completion of all interviews. Thematic saturation was assessed retrospectively and the team determined that additional interviews were not required. Data analysis was conducted using MAXQDA 2024 (VERBI Software, 2023).

### Research team and reflexivity [19]

The research team consisted of a transplant surgeon (V.R.), a senior qualitative health services researcher (J.J.), and a PhD candidate in healthcare quality and patient safety with extensive training in qualitative research methods for health services research (C.I). All interviews and focus groups were facilitated by C.I. and none of the interviewees had an established relationship with the interviewer. The combination of clinical and non-clinical perspectives of the research team helped to minimize discipline-specific bias. The transplant surgeon provided clinical insight into kidney transplant care which informed the interview guide and interpretation of findings. The senior qualitative researcher with expertise in pain management research but without transplant-specific expertise oversaw the data collection and analysis. Iterative discussions with the research team helped ensure that interpretation of the findings remained grounded in the participants’ accounts.

### Ethics and reporting

This study was conducted in accordance with the Declaration of Helsinki and was reviewed and approved by the Northwestern University Institutional Review Board (Study ID: STU00221230). This study is reported in accordance with the COnsolidated criteria for REporting Qualitative research (COREQ) Checklist [20]. 

## Results

In total, 23 clinicians participated in the qualitative study. Nine clinicians participated in individual interviews including five APPs who worked across the inpatient and outpatient settings, three attending surgeons, and one pharmacist. Seven nurses and two APPs participated in a focus group and five fellows participated in a second trainee focus group. None of the clinicians that we recruited declined to participate.

We identified six overarching themes: (1) clinicians report prescribing opioids to most KTRs as outlined in the standardized postoperative order set; (2) there is provider variation in opioid and non-opioid prescribing beyond the initial order set; (3) pain management requires a balance of setting realistic expectations about postoperative pain and controlling pain sufficiently for KTRs to complete activities of daily living; (4) clinicians believe that it is feasible to implement a protocol as long as it is flexible enough to individualize pain management based on patient-reported level and type of pain; (5) an acceptable protocol for KTRs includes preoperative blocks, scheduled acetaminophen, additional non-opioid strategies (e.g., ice, lidocaine patches, muscle relaxants, and gabapentin) and reserves oxycodone for breakthrough pain; (6) barriers to implementing a pain management protocol include concerns about inadequate pain control, delays in treatment, limited resources for preoperative blocks, and challenges changing clinician behaviors.

### Theme 1: Clinicians report prescribing opioids to most KTRs as outlined in the standardized postoperative order set

Interviewees described how pain management immediately after surgery was standardized through an order set in the electronic medical record. They stated that the current postoperative order set included hydromorphone, hydrocodone/acetaminophen, and acetaminophen as needed. The interviewees, especially the inpatient APPs, described how they tried to discontinue the intravenous hydromorphone on postoperative day one and manage pain with only the oral agents. One of the APPs who had been at the institution for many years described this as a positive change, as they had previously used more intravenous pain medications and more patient-controlled analgesia, but now use a “primarily pill-based approach.” The APPs and nurses described how they regularly reassess KTRs’ pain and work to deescalate pain medications as appropriate. One of the APPs described their overall, standardized approach to initial postoperative pain management.

Right now we have an order set. So most of our patients will get the same postop pain medications prescribed to them initially. Postop day 0…they get [hydromorphone] 0.2 every 2 h, and usually we discontinue that on postop day 1…And then we also give them [hydrocodone/acetaminophen], the 5 mg tablet, and that’s every 4 h. And then they have [acetaminophen] schedule or as needed.-Inpatient APP (Participant 4).

### Theme 2: Provider variation in opioid and non-opioid prescribing

While there was a standardized postoperative order set, there was reported variation between prescribers in opioid and non-opioid pain management strategies. Much of the variation described was within classes of medications. For example, prescribers stated preferences for cyclobenzaprine or methocarbamol for muscle relaxants. In terms of opioids, there was a lot of discussion about hydrocodone/acetaminophen compared to oxycodone. Some voiced a preference for hydrocodone/acetaminophen because of a perceived lower risk of misuse. Interviewees also described sometimes switching KTRs from hydrocodone/acetaminophen to oxycodone if pain was not well controlled but stated that this practice was not standardized. Many described that they used hydrocodone/acetaminophen because it was part of the current postoperative order set but preferred oxycodone because of the ability to increase the dose of acetaminophen. As one of the fellows described:

I invariably would always have people on oxycodone so that then I could maximize their [acetaminophen] use around the clock schedule, so they’ll always have something to provide them.- Fellow (Participant 22).

Other variations in pain management practices included the use of non-opioid strategies. Some prescribers regularly used gabapentin whereas others did not. Other variations included the use of lidocaine patches and ice. Surgeons and APPs described how their training and past experiences shaped their choices which led to variation between prescribers. This was especially common amongst the surgical fellows who described how their residency training at previous institutions influenced their current practices.

In response to the current variation in practices, many interviewees described how they would appreciate a more standardized approach through a pain management protocol.

I think consistency and standardizing would be helpful across the board. There’ll be less variation…There wouldn’t be questions from other providers, or outside, or what’s going on, as we all follow the same kind of treatment regimen. I don’t see any cons to it really.- Inpatient APP (Participant 5).

### Theme 3: Balance of expectation setting and pain control

Clinicians discussed that effective pain management requires finding a balance of setting realistic expectations with KTRs about postoperative pain and using multimodal strategies to reduce pain sufficiently to aid in recovery. All interviewees described patient education as a critical component of pain management. This education included how to safely use medications, potential side effects of medications, and expectations about pain and symptoms during recovery. In setting expectations, one of the surgeons described the role of patient education and correcting misconceptions that there should be no pain after surgery.

You kind of set the expectation a little bit. I’ll say, it’s not going to be unbearable. We’ll give you stuff to make sure it’s not unbearable. But you’re going to have some pain and you’re just going have to be okay with that.- Surgeon (Participant 7).

In terms of controlling pain sufficiently to facilitate effective recovery, several clinicians described the need to ensure that pain was controlled adequately to allow KTRs to ambulate, sleep, and eat. Most interviewees described movement and mobilization as critical to recovery and pain management.

### Theme 4: Pain management protocol needs to be individualized for each KTR

While clinicians were amenable to a standardized pain management protocol, they also emphasized the need to individualize pain management strategies. Many interviewees discussed the need to individualize prescribing to respond to KTRs’ level and type of pain. Several described their approach for assessing pain as going beyond a single measure (e.g., pain on a scale of 1 to 10), and instead asking open ended questions to assess the location, nature, and characteristics of the pain. As one of the APPs described:

My method of approaching pain control is first trying to, assess what type of pain is it? Is it a visceral pain? Is it nerve, pain? Nociceptive pain? Muscular?- Outpatient APP (Participant 3).

They then went on to describe how the type of pain guides the treatment:

That’ll help guide my diagnosis. Because for me it’s not just like they’re having pain, so we’ll have to treat it. Where is the pain coming from? And maybe if there’s a root cause that can be reversed too. Because sometimes, if it is an acute post-surgical patient, and they’ve got like a large subcutaneous hematoma, sometimes it’s as simple as draining the hematoma at the bedside to help release some of the tension and pressure.– Outpatient APP (Participant 3).

In addition to treating different types of pain differently, interviewees also discussed the need to personalize prescribing based on KTRs’ individual histories and characteristics- including allergies, response to medication, complications, and chronic pain. The clinical pharmacist stated:

I think maybe one thing we want to think about is patients [who] have had complications- maybe they have a drain in place or maybe they have other reasons related to the surgery for them to have additional pain outside of their standard surgical pain. And maybe we want to consider excluding them or working that into this kind of protocol in some way. [It cannot be] a one size fits all. All the patients are so different.-Pharmacist (Participant 2).

### Theme 5: Components of an acceptable protocol

During the interviews, clinicians were shown a draft pain management protocol for KTRs and asked to comment on their perceptions and desired changes. The draft protocol included preoperative block, scheduled acetaminophen, gabapentin, and oxycodone. It also outlined the need for patient education, daily assessment of pain scores and opioid utilization, and to ensure depression and anxiety medications were restarted and symptoms were controlled. Interviewees largely found the draft protocol to be acceptable and especially liked the use of preoperative blocks and scheduled acetaminophen. While earlier in the interviews many had discussed the tradeoffs of hydrocodone/acetaminophen and oxycodone, when viewing the protocol, all found the scheduled acetaminophen with oxycodone for breakthrough pain to be an acceptable approach. One of the outpatient APPs responded to the draft protocol stating:

I like that the gabapentin is started very low [going up to] 300 milligrams TID. So… we have the acetaminophen scheduled and Gabapentin. And I think that that’s reasonable. If they have required some opioid pain medications prior to discharge, sending them home with a short course.- Outpatient APP (Participant 3).

Questions and desired changes to the protocol included concerns about dosages of gabapentin and acetaminophen, plans for discontinuing pain medications, and availability of oxycodone. Regarding gabapentin, there were concerns about appropriate dosing given the variation in kidney function and creatinine clearance post-transplant. Others discussed how some KTRs were on gabapentin before transplant to treat chronic pain or diabetic neuropathy and questioned if the protocol would align with their preoperative doses. Some were also concerned that gabapentin could be sedating, especially for older adults. Regarding acetaminophen, some were concerned with the 4,000 mg total daily dose, stating that they were trained to not exceed 3,000 mg. One of the nurses was also concerned about the timing of the scheduled acetaminophen, stating it could cause them to need to wake KTRs up in the middle of the night. Some interviewees also requested a plan for tapering off the scheduled acetaminophen and gabapentin. Lastly, there were some questions about wanting to ensure that there was a standing order for oxycodone to avoid delays. One participant also wondered if five days of post-discharge oxycodone was enough or if it should be increased to seven days to align with the first post-transplant follow-up visit.

### Theme 6: Barriers to implementation of the pain management protocol

Clinicians shared barriers that need to be addressed during implementation of a pain management protocol for KTRs. The most commonly described barrier was the concern about timely response to breakthrough pain. Without a standing order for opioids, some of the nurses were concerned they would not be able to provide adequate pain relief to KTRs in a timely manner, especially overnight. This was especially noted as important because the hospital was a teaching hospital with rotating surgeons and trainees. As one nurse stated:

My only concern with this is that a lot of the kidneys come out during the nighttime and resources are limited overnight. So making it very clear to the resident that it’s okay to prescribe narcotics if needed. Because a lot of times there’s just a chain. They don’t want to prescribe the narcotics, so they have to check with the fellow. That took them 30 minutes to do. Then the fellow has to tell them to put in the order that then has to get verified by pharmacy and at this point we’re two hours from when we started this process and it’s hard to get them the medication.-Nurse (Participant 10).

The concerns about adequate pain controlled extended to the outpatient setting where some of the interviewees were worried about additional follow-up phone calls, emergency department visits, and readmission for uncontrolled pain.

Inevitably you always worry about Saturday at 3:00 AM they need it. They can’t get ahold of anyone. Pharmacies are closed.-Outpatient APP (Participant 1).

While all interviewees viewed preoperative blocks positively, many shared concerns about the availability of resources to consistently utilize these. This was especially true for deceased donor transplants where timing was unpredictable, and anesthesia resources were limited overnight. One of the fellows described this as *“the barrier is usually timing”* with another adding they would proceed without a preoperative block *“if it’s going to hold us up.”* They voiced the need for streamlined communication with the regional anesthesia team to facilitate consistent use of preoperative blocks.

The final concern was related to the perception that a pain management protocol, particularly one that was opioid-sparing, would not meet the needs of all KTRs. While some interviewees were supportive of moving toward opioid-free strategies, most interviewees felt that at least a short course of opioids was needed for most KTRs. The concerns were primarily around the variation in how KTRs experience pain and wanting to have options readily available. One of the outpatient APPs described this as:

I think there’s such variability. I mean pain is so subjective. We know that. Some patients never take narcotics and do fabulous, and then other patients are taking narcotics or something every 4 h, and still rating their pain as an 8. So I think non opioid is great, is a great goal. And I mean I like it. I think a lot of patients will do well with it, but I think there always has to be an option, for you know, patients that need it because there are going to be patients that need it. Outpatient APP (Participant 6).

## Discussion

Through interviewing clinicians about their current pain management practices, we found that there was reliance on opioids for pain management and variation in practice beyond an initial post-operative order set. Clinicians were enthusiastic about the potential of implementing a pain management protocol, as long as the protocol was flexible enough to individualize prescribing to each KTR’s unique needs. Implementation of such a protocol would require addressing identified barriers including availably of resources for preoperative blocks and workflow to ensure timely prescribing of medications for breakthrough pain.

There is an ongoing movement to improve pain management strategies and reduce the use of opioids in solid organ transplantation. A systematic review of the literature found consistent evidence for the negative, dose-dependent effect of opioids on mortality and graft failure [21]. This review also highlighted the value of the multimodal protocols that combine scheduled acetaminophen, gabapentin, lidocaine patches, transversus abdominis plane/quadratus lumborum blocks, ketamine, cessation of patient-controlled analgesia, targeted education, and selective opioid ordering to reduce opioid use [21]. Various opioid reductions initiatives following similar outlines have been successful at reducing opioid prescribing and opioid use in kidney transplant [11, 12, 14]. However, these practices are still not routine, and implementation work is needed to ensure consistent application.

Effective implementation of opioid reduction initiatives requires careful consideration of the local processes, barriers, and facilitators. Studies across disciplines of surgery have found consistent barriers to implementing opioid reduction initiatives. Time and resources, modality of educational material, and concerns over patient satisfaction scores have previously been identified as barriers [22]. An implementation evaluation of a large opioid reduction initiative in surgery found high variability in response to the intervention, the need to view surgical pain management as a team effort, and the impact of prior established believes about opioid risks [23]. Others have described perceived barriers as the restricted ability to electronically prescribe controlled substances, coverage during nights and weekends, lack of time for patient education, and financial and logistical barriers to non-opioid pain management strategies [24]. This current study highlights many of the same challenges in the setting of kidney transplantation and highlights the importance of addressing clinicians’ concerns before implementing a new care process.

Our study highlights the importance of understanding local context before implementation of a new intervention. Different settings (e.g., non-academic or lower-resource transplant centers) will likely have different current practices and logistic constraints that need to be accessed prior to implementing a pain management protocol. Centers planning to implement pain management protocols should consider potential barriers and contextual factors before implementation to maximize the chances of success. First, it is important to gain an understanding of current practices and assess variation in practice before implementation and co-design a protocol that is acceptable to clinicians across the care team. Second, as described by participants in our study, it is important to ensure that a pain management protocol is flexible enough to adapt to individual patients’ level and type of pain and manage pain with multimodal strategies. Third, centers should assess their processes before implementation of a pain management protocol to prevent delays in treatment of breakthrough pain and ensure adequate support for preoperative blocks.

This study has a few limitations which should be considered in interpreting the results. First, this is a single-center study from a large, urban, academic transplant center. The findings might therefore be different in other transplant centers with different characteristics. While the goal of qualitative research is not generalizability in the statistical-probabilistic sense, transferability occurs when a person or group in one setting considers adopting something from another that the research has identified [25]. The findings from this study can therefore be transferable to other settings where they can be adapted based on local context. Second, although we included a diverse sample of clinicians- including surgeons, surgical trainees, APPs, nurses, and a pharmacist- we did not interview all relevant stakeholders including patients or anesthesia. Future research which includes patients will be critical to understand patient perceptions of pain management protocols and opioid minimization to assure that interventions improve safety without decreasing patient satisfaction. Anesthesiology will also need to be engaged for future interventions for pain management, especially related to preoperative blocks. Third, because of the voluntary nature of research, it is possible that our sample is biased towards individuals with greater interest in opioids minimization or process improvement. Fourth, social-desirability bias, or the tendency for participants to respond in a manner that will be viewed favorably by others, could have influences responses, especially in the focus groups. To mitigate this, we ensured confidentiality, reminded participants that there were no right or wrong answers, and encouraged them to follow up individually with any additional comments or concerns. Fifth, all data was collected and coded by a single researcher. Credibility and trustworthiness were maintained through iterative team review of the data and emerging findings, through a team with both clinical and non-clinical expertise.

Overall, our qualitative study shows that clinicians across disciplines are supportive of a pain management protocol for KTRs, provided it is flexible and able to be tailored to meet individual patient needs. Implementation of pain management protocols need to attend to the local facility needs -- in this case ensuring timely access to prescribing for breakthrough pain and enhanced communication and coordination with anesthesiology for preoperative blocks.

## Electronic Supplementary Material

Below is the link to the electronic supplementary material.

Supplementary Material 1

## Data Availability

The data that support the findings of this study are not openly available due to reasons of sensitivity and are available from the corresponding author upon reasonable request.

## Abbreviations

APP: Advanced practice provider
KTR: Kidney transplant recipient
US: United States of America

## References

[1] U.S. Overdose deaths involving prescription opioids, 1999–2023: National institute on drug abuse [Available from: https://nida.nih.gov/research-topics/trends-statistics/overdose-death-rates#Fig4.

[2] Florence C, Luo F, Rice K. The economic burden of opioid use disorder and fatal opioid overdose in the United States, 2017. Drug Alcohol Depend. 2021;218:108350. 33121867 10.1016/j.drugalcdep.2020.108350 PMC8091480

[3] Hill MV, McMahon ML, Stucke RS, Barth RJJ. Wide Variation and Excessive Dosage of Opioid Prescriptions for Common General Surgical Procedures. Ann Surg. 2017;265(4):709–14. 27631771 10.1097/SLA.0000000000001993

[4] Substance abuse and mental health services administration. Key substance use and mental health indicators in the United States: results from the 2024 National Survey on drug use and health (HHS Publication No. PEP25-07-007, NSDUH Series H-60). Substance abuse and mental health services administration. 2025.

[5] Brummett CM, Waljee JF, Goesling J, Moser S, Lin P, Englesbe MJ, et al. New Persistent Opioid Use After Minor and Major Surgical Procedures in US Adults. JAMA Surg. 2017;152(6):e170504–e. 28403427 10.1001/jamasurg.2017.0504 PMC7050825

[6] Wise B, Wilson LZ, Taber DJ, Pilch NA, Rohan V, Fleming JN. The impact of pretransplant opioid exposure on healthcare utilization and costs in kidney transplant. Pharmacotherapy: J Hum Pharmacol Drug Therapy. 2021;41(1):6–13. 10.1002/phar.2479 33107627

[7] Abbott KC, Fwu C-W, Eggers PW, Eggers AW, Kline PP, Kimmel PL. Opioid Prescription, Morbidity, and Mortality in US Transplant Recipients. Transplantation. 2018;102(6):994–1004. 29319627 10.1097/TP.0000000000002057 PMC5962376

[8] Lentine KL, Lam NN, Naik AS, Axelrod DA, Zhang Z, Dharnidharka VR, et al. Prescription opioid use before and after kidney transplant: Implications for posttransplant outcomes. Am J Transplant. 2018;18(12):2987–99. 29498196 10.1111/ajt.14714 PMC6119653

[9] Muir MA, Szempruch KR, Dupuis R, Toledo AH, Isaak RS, Arora H, et al. Utilizing multimodal analgesia to evaluate postoperative analgesic requirements in kidney transplant recipients. Clin Transplant. 2021;35(4):e14240. 33525058 10.1111/ctr.14240

[10] Dualeh SHA, McMurry K, Herman AE, Maryan S, Pacurar LA, Waits SA, et al. Evaluation of an opioid restrictive pain management initiative in adult kidney transplant recipients. Clin Transplant. 2021;35(7):e14313. 33838060 10.1111/ctr.14313

[11] Rohan VS, Taber DJ, Patel N, Perez C, Pilch N, Parks S, et al. Impact of a multidisciplinary multimodal opioid minimization initiative in kidney transplant recipients. Clin Transplant. 2020;34(10):e14006. 32524643 10.1111/ctr.14006

[12] Carcella T, Patel N, Marable J, Bethi S, Fleming J, Baliga P, et al. Long-term Outcomes Following a Comprehensive Quality Assurance and Process Improvement Endeavor to Minimize Opioid Use After Kidney Transplant. JAMA Surg. 2023;158(6):618–24. 37017945 10.1001/jamasurg.2023.0276 PMC10077134

[13] Schwab ME, Braun HJ, Ascher NL, Hirose R. Implementing an opioid reduction protocol in renal transplant recipients. Am J Surg. 2020;220(5):1284–9. 32650975 10.1016/j.amjsurg.2020.06.055 PMC9129056

[14] Schwab ME, Braun HJ, Quan D, Roll GR, Budanova N, Ascher NL, et al. Standardizing Discharge Opioid Prescriptions in Kidney Transplant Patients Decreases Opioid Usage. J Surg Res. 2021;265:153–8. 33940238 10.1016/j.jss.2021.03.038

[15] Bova S, Samet RE, Deering J, Gaines S, Weinrub A, Bhati C, et al. Successful opioid minimization following kidney transplant: a quality improvement initiative. Cureus. 2024;16(1). 10.7759/cureus.52917 PMC10896457 38410295

[16] Kim H, Jarrett JB, Ingemi AI, Lichvar A. Opioid use practices of kidney transplant centers in the United States: A national survey of institutional practices. JACCP: J Am Coll Clin Pharm. 2021;4(5):587–93.

[17] Hagaman A, Rhodes EC, Aloe CF, Hennein R, Peng ML, Deyling M, et al. How Are Qualitative Methods Used in Implementation Science Research? Results From a Systematic Scoping Review. Implement Res Pract. 2025;6:26334895251367470. 40894413 10.1177/26334895251367470 PMC12394868

[18] Hamilton AB, Finley EP. Qualitative methods in implementation research: An introduction. Psychiatry Res. 2019;280:112516. 31437661 10.1016/j.psychres.2019.112516 PMC7023962

[19] Creswell JW, Báez JC. 30 essential skills for the qualitative researcher. Sage. 2020.

[20] Tong A, Sainsbury P, Craig J. Consolidated criteria for reporting qualitative research (COREQ): a 32-item checklist for interviews and focus groups. Int J Qual Health Care. 2007;19(6):349–57. 17872937 10.1093/intqhc/mzm042

[21] Kutzler HL, Lichvar AB, Quan D, Bowman LJ, Diamond A, Doligalski C, et al. A systematic review of opioid use and multimodal strategies in solid organ transplant recipients and living donors. Pharmacotherapy: J Hum Pharmacol Drug Therapy. 2023;43(6):514–51. 10.1002/phar.2808 37157142

[22] Coughlin JM, Shallcross ML, Schäfer WLA, Buckley BA, Stulberg JJ, Holl JL, et al. Minimizing Opioid Prescribing in Surgery (MOPiS) Initiative: An Analysis of Implementation Barriers. J Surg Res. 2019;239:309–19. 30908977 10.1016/j.jss.2019.03.006 PMC8527839

[23] Schäfer WLA, Johnson JK, Ager MS, Iroz CB, Huang R, Balbale SN, et al. Learning from the implementation of a surgical opioid reduction initiative in an integrated health system: a qualitative study among providers and patients. Implement Sci Commun. 2024;5(1):22. 38468284 10.1186/s43058-024-00561-4 PMC10926556

[24] Sceats LA, Ayakta N, Merrell SB, Kin C. Drivers, Beliefs, and Barriers Surrounding Surgical Opioid Prescribing: A Qualitative Study of Surgeons’ Opioid Prescribing Habits. J Surg Res. 2020;247:86–94. 31767277 10.1016/j.jss.2019.10.039

[25] Smith B. Generalizability in qualitative research: misunderstandings, opportunities and recommendations for the sport and exercise sciences. Qualitative Res Sport Exerc Health. 2018;10(1):137–49.

