# Supplementary material for: Clinician perspectives on the feasibility of a pain management protocol in kidney transplant
Source: BMC Nephrol. 2026 May 22;27:431. doi: 10.1186/s12882-026-05046-1 (PMC13374193; doi:10.1186/s12882-026-05046-1)

**Interview/Focus Group Protocol**

**Interviewees:** transplant surgeons, fellows, inpatient and outpatient Advanced Practice Providers (APPs), pharmacists, post anesthesia care unit (PACU) nurses, and floor nurses

Introduction: Thank you for agreeing to talk with us today. We are working on a project to standardize opioid prescribing and pain management for patients undergoing kidney transplant here at NM. We are conducting interviews with several people to learn about your current practices for pain management and perspectives on how to optimize care for our patients. Your interview transcript will be de-identified, so your specific comments will not be attributed to you.

[Verbal consent]

We would like to record the interview because that makes it easier for us to focus on the conversation instead of taking notes. Is it okay with you if we record our conversation?

Do you have any questions for us before we start?

______________________________

**Current Practices**

1. Can you tell us about how you manage pain for kidney transplant patients?
   1. What are your current opioid prescribing practices for kidney transplant patients?
   2. How do you determine the **amount and type of pain medication** to prescribe?
      1. Is this **standard** or does it vary between patients?
      2. What type and quantity of **opioids** do you prescribe?
   3. Do you prescribe or recommend any **non-opioid** pain management strategies?
      1. If so, what are they?
   4. What type of **education** do you provide to patients regarding postoperative pain management?
   5. How do you handle patient requests for **refills** of opioid medications?
2. Walk me through the process for prescribing opioid pain medication for kidney transplant patients.
   1. Who is involved and what are their roles?

**Feasibility**

1. How would you feel about following a standard protocol for prescribing opioids?
2. What do you think will be the barriers, if any, to standardizing pain management for kidney transplant patients?
   1. How supportive do you think **senior leadership** is of this intervention?
   2. How supportive do you think your **peers** are of this intervention?
   3. How do you think your **patients** will respond to this intervention?
   4. What are your thoughts about **how to address** [specific barrier(s) mentioned by participant]?
   5. What kind of **supporting evidence or information** is needed about the effectiveness of the intervention components to get staff on board? *[CFIR Domains – relative priority (implementation climate, inner setting), evidence strength and quality (intervention characteristics)]*
   6. What kinds of educational materials and resources do you think would be most helpful for providers and patients? What **additional resources or tools** do you think would be helpful?
3. Are you aware of any other current efforts to reduce or standardize opioid prescribing for kidney transplant patients? If yes, please elaborate.

**Adaptations Needed**

1. [Show Draft Pain Management Protocol] Some other centers use standardized pain management protocols. Here is an example of one of those protocols. We are curious about how this might work here at NM and want to hear your perspective on changes that should be made.

**Conclusion:**

Is there anything else that you would like to tell us about today?

Thanks for agreeing to take time to meet with us today.

Draft Pain Management Protocol- Presented to interviewees for their feedback


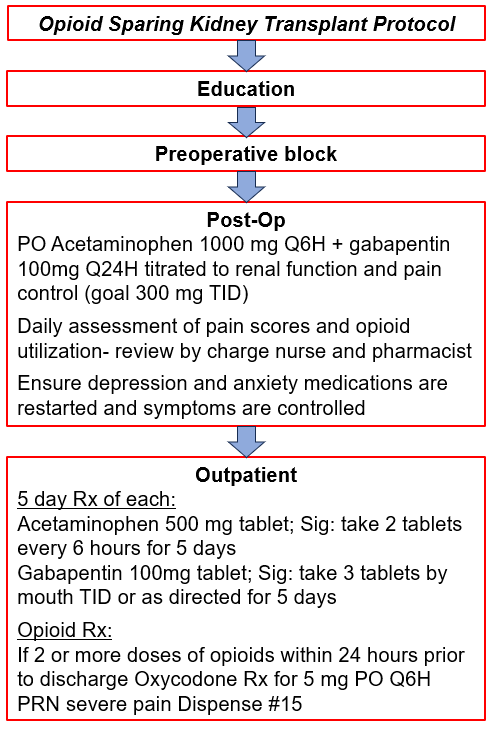

Supplement: Supplementary file 1 — Supplementary Material 1 [file 12882_2026_5046_MOESM1_ESM.docx]
